# Weight loss practice, nutritional status, bone health, and injury history: A profile of professional jockeys in Korea

**DOI:** 10.20463/jenb.2018.0021

**Published:** 2018-09-30

**Authors:** Soeun Jeon, Kyungho Cho, Gina Ok, Sukho Lee, Hyon Park

**Affiliations:** 1 Department of Sports Medicine, Kyung Hee University, Yongin Republic of Korea; 2 Department of Counseling, Health, and Kinesiology, Texas A&M University-San Antonio, Texas USA

**Keywords:** jockeys, extreme weight loss, nutritional assessment, bone mineral density, injury

## Abstract

**[Purpose]:**

The purpose of this study was to investigate the impact of weight loss practices on nutritional status, bone health, and injury history among Korean professional jockeys.

**[Methods]:**

Forty-three male jockeys completed a questionnaire to assess their weight loss practices. Of these, 10 jockeys were selected for in-depth assessment of their nutritional status, bone health, and injury history.

**[Results]:**

The questionnaires revealed that 81.4% of jockeys lost weight every week mainly by dieting and/or exercising. None of the jockeys consumed enough food during the weight loss period. Two jockeys were diagnosed with osteopenia and one was diagnosed with osteoporosis. Only history of fracture showed a significant correlation with low bone mineral density. All jockeys had more than one injury experience throughout their career. Fracture was the most common type of injury, occurring during practice and/or competition and caused mainly by difficulties in handling the horses.

**[Conclusion]:**

Professional jockeys in Korea use extreme weight loss methods. Their repeated periods of poor nutritional intake may result in seriously low bone mineral density, which may aggravate injuries sustained during horse races. Implementation of balanced dietary programs and delivery of health education on weight management are urgently required.

## INTRODUCTION

Horse racing is one of the oldest sports in the world. The speed of the horse is key in competition. To achieve maximal speed in racehorses, the body weight of jockeys must be less than 49 kg in Korea (2016 Guideline for Korean Trainee Jockeys of the Korea Racing Authority (KRA)). Many jockeys endeavor to comply with the strict weight restrictions before each competition by adopting unhealthy weight loss practices that are relatively quick, such as restricting food and fluid consumption, taking laxatives or diuretics, and even vomiting after meals^1–6^. Of concern is the issue that racing competitions take place every weekend all year round, except for one month during the off season. Therefore, jockeys repeatedly engage in these unhealthy weight loss practices throughout the year.

Unhealthy weight loss practices can negatively affect the health condition of jockeys. A poor diet cannot properly fulfil the nutritional intake required for the human body to function and have good performance^7–17^. Dehydration caused by restricting fluid intake or promoting perspiration can decrease total plasma volume in the body, which has adverse effects on overall physiological functions including hormonal imbalance, kidney and cardiovascular dysfunction, electrolyte imbalance, and impaired thermoregulation^11,13,18–21^. In addition, repeated unhealthy weight loss practices lead to a lack of vital mineral and vitamin intake that can persist for many years. This insufficient nutrient intake hampers the maintenance of proper bone mineral density (BMD)^12,14,22–25^. Relatively low BMD among professional jockeys has been reported in numerous studies^2,4,5,26–28^.

Not only physiological functions but also psychological and cognitive functions are negatively influenced by extreme dietary regimens^9,11–13,15^. Previous studies have reported that jockeys who undergo rapid weight loss sometimes feel fatigue, confusion, a lack of concentration, and experience depression^1,4,29–31^. A marked decline in cognitive function may distract their attention during horse racing, which increases the possibility of falling during competition.

Falling accidents happen frequently in horse racing, and even death can occur in the worst case^32–37^. In general , jockeys have relatively low BMD, which is not only likely to cause more severe injury if a fall occurs, it also significantly influences jockeys’ overall health, even after their professional racing career is over^32^.

Despite the fact that jockeys consistently practice unhealthy weight loss strategies, there is no systematic education and management of weight control practices for this population in Korea. Therefore, the purpose of this study was to investigate the impact of weight loss practices on nutritional status, bone health, and injury history among Korean professional jockeys who often engage in extreme weight loss. We expect to provide baseline data regarding Korean professional jockeys through this study.

## METHODS

### Participants

Forty-three professional male jockeys from the KRA completed a questionnaire on their weight loss practices. Of these, we selected 10 jockeys who reported using extreme weight loss practices for more than 5 years, to examine their nutritional status, bone health, and injury history in greater detail. Prior to starting our study, all participants were given a full explanation of all procedures, and they provided their written informed consent. This study was approved by the Texas A&M University-San Antonio Institutional Review Board (2017-22).

### Weight loss practices questionnaire

In this study, we used a modified weight loss practices questionnaire, adopted from previous studies^1,38^. This modified questionnaire contained 13 open-and closed-ended questions on general characteristics, weight loss practices, psychological and physiological changes after weight control, and recovery methods. Some questions had more than one possible answer. The questionnaire was self-administered by the respondents.

### Anthropometric and body composition assessment

Height, body mass, and total body water were measured using a multi-frequency bioelectrical impedance analysis (BIA) device (X-SCAN PLUS 2; Jawon Medical, Korea). Body mass index (BMI) was calculated as weight divided by height squared (kg/m^2^). Lean and fat mass and percent body fat were assessed using dual-energy X-ray absorptiometry (DEXA) (QDR-4500; Hologic, USA).

### Nutritional assessment

Jockeys' nutritional status was assessed on two different days (weight loss day vs. non-weight loss day) using a 24-hour dietary recall. All participants filled out dietary logs after being provided with detailed instructions, and a researcher reviewed the logs together with each jockey. The data were analyzed using a computer-aided nutritional analysis program (CAN-Pro 2.0; The Korean Nutrition Society, Korea). The estimated average requirement (EAR) and recommended daily allowance (RDA) were based on the Dietary Reference Intakes for Koreans in 2015.

### Bone health

BMD and bone mineral content (BMC) of the total body were assessed using DEXA scans (QDR-4500). BMD was reported as grams of absolute BMC per cm^2^. Osteopenia and osteoporosis were based on the criteria of the World Health Organization. Osteopenia was defined as T-score between −1.0 and −2.5, and osteoporosis was defined as T-score of −2.5 or lower. To analyze the correlation between BMD and osteoporosis risk factors, data on previous fracture history, smoking, and alcohol consumption were collected using a separate questionnaire^22,39^.

### Injury history questionnaire

The injury history questionnaire contained nine open-and closed-ended questions on the number of injuries experienced, time and cause of injury, body sites of injury and diagnosis, therapeutic period, number of recurrences, and degree of influence on competition^32,33,36^. All questions, except for the number of injuries, had more than one possible answer. The questionnaire was self-administered by respondents.

### Statistical analysis

All data are presented as mean ± standard deviation and were analyzed with IBM SPSS Statistics 24 (IBM Corp., Armonk, NY, USA). Frequency analysis was performed for data collected using the weight loss practices questionnaires. Jockeys’ nutritional status between weight loss days and non-weight loss days were compared using paired t-tests, with effect sizes calculated as Cohen’s drm^40^. Spearman’s rank correlation coefficient was used to test the association between BMD and osteoporosis risk factors: age, fracture history, smoking, alcohol consumption, calories and calcium consumption on weight loss days, and calories and calcium consumption on non-weight loss days. Statistical significance was set at *p* < .05.

## RESULTS

### Weight loss practices questionnaire

Forty-three professional jockeys responded to the weight loss practices survey (age 32.3 ± 7.2 years, career 10.5 ± 7.8 years, height 158.3 ± 4.8 cm, body mass 49.8 ± 2.3 kg). More than 80% of jockeys underwent a routine weight loss regimen every week, 1 and 3 days prior to race days, mainly by extreme dieting, exercising, and/or using a sauna. Around 70% of jockeys reported severe fatigue during weight loss days. They consumed high caloric foods and/or rested to recover their condition after each competition (Table 1).

**Table 1.** Results of weight loss practices questionnaires among Korean professional jockeys.

n (%)		n (%)	
Weight control status (n=42)		Psychological changes after weight loss (n=35)	
Trying to lose weight	35 (81.4)	Irritation	22 (62.9)
Not trying to lose weight	8 (18.6)	No change	7 (20.0)
Number of weight loss days per week (n=35)		Anger	
1 day	11 (31.4)	Depression	5 (14.3)
2 days	10 (28.6)	Anxiety	3 (8.6)
3 days	12 (34.3)	Tiredness	3 (8.6)
More than 4 days	3 (8.6)	Physiological changes after weight loss (n=35)	
Amount of weight usually lost (n=35)		Fatigue	25 (71.4)
Less than 1 kg	25 (71.4)	Hunger	21 (60.0)
1 to 2 kg	7 (20.0)	Thirst	18 (51.4)
More than 2 kg	3 (8.6)	Muscle cramps	8 (22.9)
Weight loss methods (n=35)		Dizziness	7 (20.0)
Restricting calories	22 (62.9)	Dehydration	4 (11.4)
Increased exercise	21 (60.0)	No change	2 (5.7)
Sauna	18 (51.4)	Recovery methods after competitions (n=35)	
Fasting	9 (25.7)	Consuming calories	33 (94.3)
Restricting fluids	7 (20.0)	Resting	23 (65.7)
Vomiting after meals	2 (5.7)	Nutritional supplements	8 (22.9)
Diuretics or laxatives	0 (0.0)	Other	4 (11.4)

### Anthropometric and body composition

Anthropometric and body composition data of the 10 jockeys is presented in Table 2.

**Table 2.** Anthropometric and body composition profiles of Korean professional jockeys.

	Jockeys (n = 10)
Age (years)	31.8±3.7
Career (years)	11.6±3.8
Height (cm)	157.5±4.52
Body mass (kg)	50.6±1.87
BMI (kg/m2)	20.5±1.38
Lean body mass (kg)	43.3±1.67
Fat mass (kg)	7.3±1.24
Percent body fat (%)	14.4±2.27
Total body water (kg)	31.2±1.20

Note. Data presented as mean ± SD.

### Nutritional status

The results of nutritional assessment are presented in Table 3. On weight loss days, jockeys not only consumed around half the calories and EAR consumed on non-weight loss days, they also did not eat the RDA of micronutrients. Most nutrients were consumed in significantly lower amounts during weight loss days compared with non-weight loss days: total calories (*t*_(9)_ = −5.09, *p* < .001), carbohydrate (*t*_(9)_ = −2.93, *p* = .017), protein (*t*_(9)_ = −4.01, *p* = .003), fat (*t*_(9)_ = −3.08, *p* = .013), vitamin E (*t*_(9)_ = −2.81, *p* = .020), thiamine (*t*_(9)_ = −3.48, *p* = .007), riboflavin (*t*_(9)_ = −2.53, *p* = .032), niacin (*t*_(9)_ = −3.91, *p* = .004), vitamin B6 (*t*_(9)_ = −3.59, *p* = .006), phosphorus (*t*_(9)_ = −3.84, *p* = .004), sodium (*t*_(9)_ = −5.90, *p* < .001), potassium (*t*_(9)_ = −2.73, *p* = .023), and zinc (*t*_(9)_ = −4.59, *p* = .001).

**Table 3.** Nutritional analysis of Korean professional jockeys.

	Weight loss days	n (%) < RDA	Non-weight loss days	n (%) < RDA	*d*
Total calories (kcal)	1046.0 ± 590.86		2086.5 ± 374.76***		1.65
EAR (kcal)	2806.7 ± 74.81		2806.7 ± 74.81		
Carbohydrate (g)	156.9 ± 118.76		251.6 ± 95.98*		0.95
Protein (g)	38.3 ± 18.39		89.3 ± 36.26**		1.34
Fat (g)	29.0 ± 14.59		61.8 ± 26.59*		1.00
Vitamin A (μg)	443.6 ± 367.47	8 (59.2)	883.0 ± 866.20	7 (117.7)	0.60
Vitamin E (μg)	6.3 ± 5.41		11.1 ± 5.56*		0.89
Vitamin C (mg)	59.7 ± 54.76	9 (59.7)	92.9 ± 60.55	6 (92.9)	0.41
Thiamine (mg)	0.62 ± 0.42		1.46 ± 0.84**		1.22
Riboflavin (mg)	0.61 ± 0.41		1.18 ± 0.57*		1.07
Niacin (mg)	8.1 ± 3.10	9 (50.7)	20.0 ± 8.03**	5 (124.8)	1.25
Vitamin B6 (mg)	1.2 ± 0.85	9 (62.0)	2.4 ± 0.77**	3 (120.0)	1.17
Folate (μg)	157.2 ± 132.87	10 (39.3)	220.7 ± 116.54	9 (55.2)	0.58
Calcium (mg)	266.1 ± 184.33	9 (38.0)	385.3 ± 157.56	6 (55.0)	0.63
Phosphorus (mg)	532.9 ± 307.55	8 (76.1)	1061.1 ± 394.73**	1 (151.6)	1.23
Sodium (g)	1.9 ± 0.47		4.3 ± 1.49***		2.58
Potassium (g)	1.6 ± 1.03		2.6 ± 0.94*		0.83
Iron (mg)	13.0 ± 17.69	7 (130.5)	13.8 ± 2.93	2 (138.0)	0.08
Zinc (mg)	5.2 ± 2.38	9 (54.3)	10.0 ± 2.59**	4 (105.3)	1.44

Note. Data presented as mean ± SD unless otherwise indicated.

d = Cohen’s d effect size, EAR = estimated average requirement, RDA = recommended daily allowance.

n (%) < RDA refers to the number of people who consumed less than the RDA and the average proportion of the RDA represented by the intake amount.

* p < .05, ** p < .01, *** p < .001 between weight loss days and non-weight loss days.

### Bone health

**Fig. 1.**
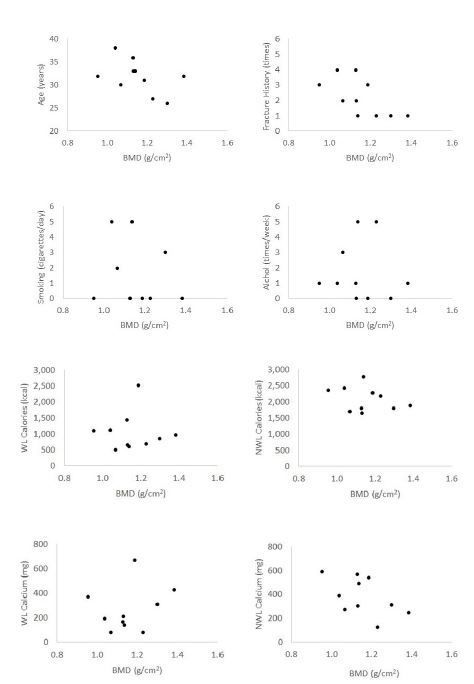
Correlation between total bone mineral density (BMD) and risk factors for osteoporosis. WL = weight loss days, NWL = non-weight loss days.

Results of DEXA scans are presented in Table 4. Two jockeys were diagnosed with osteopenia and one was diagnosed with osteoporosis. All jockeys had experienced fractures more than once (2.2 ± 1.23). Six jockeys were non-smokers, one usually smoked 6 to 10 cigarettes a day, another smoked 11 to 15 cigarettes a day, and two jockeys smoked a pack per day. As for alcohol consumption, three jockeys never drank, four drank alcohol less than once a week, one drank 2 to 3 times a week, and two jockeys drank more than 4 times a week. Figure 1 shows the Spearman's correlation coefficients between BMD and risk factors for osteoporosis. Only fracture history had a significantly strong correlation: *r*(8) = −75 , *p* = .013. Other risk factors showed no significant correlations with BMD: age (*r*_(8)_ = −.51, *p* = .136), smoking (*r*_(8)_ = −.15, *p* =.678), alcohol (*r*_(8)_ = −.13, *p* = .726), non-weight loss calories (*r*_(8)_ = −.13, *p* = .726), weight loss calories (*r*_(8)_ = −.08, *p* = .829), non-weight loss calcium (*r*_(8)_ = −54 , *p* = .108), and weight loss calcium (*r*_(8)_ = .20, *p* = .580).

**Table 4.** Mean values for bone mass among Korean professional jockeys.

	Area (cm^2^)	BMC (g)	BMD (g/cm^2^)
Left arm	159.44 ± 9.63	128.32 ± 12.53	0.804 ± 0.049
Right arm	172.59 ± 10.64	139.02 ± 13.25	0.806 ± 0.058
Left ribs	116.83 ± 11.95	80.04 ± 11.10	0.688 ± 0.092
Right ribs	119.28 ± 13.10	81.14 ± 12.71	0.679 ± 0.066
Thoracic spine	107.78 ± 14.39	97.37 ± 16.18	0.906 ± 0.102
Lumbar spine	46.90 ± 6.92	54.36 ± 17.89	1.150 ± 0.297
Pelvis	186.87 ± 22.38	194.72 ± 38.70	1.035 ± 0.090
Left leg	302.06 ± 20.28	353.62 ± 52.12	1.167 ± 0.110
Right leg	295.93 ± 22.29	353.51 ± 56.60	1.196 ± 0.114
Subtotal	1507.67 ± 67.35	1482.10 ± 186.27	0.908 ± 0.085
Head	267.19 ± 20.17	574.43 ± 129.85	2.145 ± 0.401
Total	1774.06 ± 77.59	2056.53 ± 306.71	1.155 ± 0.126
T-score		−0.29 ± 1.41	
Z-score		0.00 ± 1.35	

Note. Data presented as mean ± SD.

BMC = bone mineral content, BMD = bone mineral density.

### Injury history questionnaire

The results of the questionnaire on jockeys’ injury history throughout their career are presented in Table 5. All jockeys had experienced more than one injury. Two jockeys were injured more than 10 times. Most injuries took place during practice or competition and were mainly caused by difficulties in handling the horses. The most frequently injured body parts were the shoulder and lower back. Fractures happened most frequently, but recurrence of these injuries was rare. In most cases, jockeys were unsatisfied with the length of their recovery period and felt that their injuries negatively affected future competitions.

**Table 5.** Injury history among Korean professional jockeys.

n		n	
Number of injuries experienced		Injury of lower body site	
1 to 3 times	1	Knee	1
4 to 6 times	3	Calf	1
7 to 9 times	3	Ankle	1
More than 10 time	2	Toe	2
Time of injury		Diagnosis	
Warm-up	2	Fracture	13
Practice	15	Dislocation	5
Competition	20	Strain	3
Cause of injury		Sprain	5
Horse	29	Myositis	3
Facilities	3	Tendinosis	1
Another person	1	Herniated disc	2
Poor conditions	2	Abrasion	4
Nervousness	1	Laceration	2
Excessive competitiveness	3	Recurrence	
Ineptitude while riding the horse	2	Never	18
Injury involving upper body site		1 time	3
Head	1	More than 2 times	4
Face	3	Therapeutic period	
Neck	4	Very short	4
Shoulder	6	Short	14
Chest	3	Neutral	7
Back	2	Sufficient	8
Low back	6	Influence on competition	
Elbow	3	Not at all	3
Wrist	2	Somewhat	5
Finger	5	Definitely	15

## DISCUSSION

The aim of this study was to investigate the weight loss practices of Korean professional jockeys and to scrutinize jockeys’ characteristics including nutritional status, bone health, and injury history, targeting jockeys who had engaged in more than 5 years of extreme weight loss practices.

Our findings on the questionnaire survey for weight loss practices of jockeys were in line with those of previous studies^1–3,6,30,31,41^. We confirmed that many jockeys have extreme weight loss routines that mainly involve restricting calories and/or inducing dehydration. A large number of studies have pointed out that this eating disorder pattern, including starvation, exercise-induced dehydration, binge eating, and vomiting has a high association with numerous disorders^8,12,23^. People with eating disorders are not only more likely to have various health problems such as gastrointestinal disorders, low bone density, and hormonal problems in later life, they are less likely to reach peak athletic performance owing to low available energy^10,15^. Mental disorders, such as mood disorders, anxiety, and depression, are additional consequences of eating disorders^43,44^. For these reasons, the National Athletic Trainers’ Association (NATA) announced guidelines to manage and reduce disordered eating in athletes^45^. Because most jockeys in this study also reported one or more negative physiological and psychological changes after weight loss, finding proper ways to protect jockeys’ health in consideration of the unique requirements of their sport is urgently needed.

As reported in numerous jockey-related studies, not consuming enough of the nutrients needed for good health is typical among jockeys^1–5^. Our study also found that while losing weight, all jockeys consumed about half the calories consumed on non-weight loss days, and most nutrients consumed did not reach the EAR or RDA on both weight loss and non-weight loss days. Korean jockeys tend to start weight loss just 1 or 2 days before competition, usually by restricting food and fluid intake. In addition, jockeys cannot replenish food and water before or during competition because their weight must match before and after each race. Such low energy availability induced by extreme weight loss practices makes it hard for these athletes to focus on racing and to reach peak physical performance levels^46^. At worst, poor physical condition on the day of competition may result in falling accidents.

The negative effects of low calorie consumption can be either acute or chronic. A repetitive pattern of poor calorie intake among jockeys can enervate overall health. In Joint Position Statements , it is highly recommended that athletes consume at least the RDA of all micronutrients because low energy intakes can result in failure to achieve peak bone density and can lead to increased fatigue, injury, and illness^14^. People aged 19–70 years are recommended to intake at least 1,000 mg of calcium a day for bone health^25^, and athletes with disordered eating or at risk for early osteoporosis are recommended 1,500 mg of calcium intake a day^14^. The average daily dietary calcium intake of Korean jockeys in this study was less than half their RDA. Jockeys’ unhealthy diet regimen may arise from poor nutritional knowledge due to a lack of nutritional education for these athletes in Korea. Therefore, it is essential to improve nutritional education as a collaborative effort among nutritionists, counselors, and jockeys, to forestall adverse effects caused by chronic undernourishment in this population.

As for bone health, peak bone mass typically occurs in the early 30s^47^. According to a population-based cross-sectional analysis among 398 women and 222 men aged 20–89 years, the average total BMD of men aged 20–29 years was 1.26 ± 0.10 g/cm^2^, and the average total BMD of men aged 20–39 was 1.24 ± 0.11 g/cm^2^^47^. In comparison with this study, Korean jockeys in our study had relatively low total BMD (1.155 ± 0.126 g/cm^2^), which did not seem to reach its peak value. Similarly, Dolan et al.^26^ showed that 20 Irish professional jockeys had an average 1.134 ± 0.05 g/cm^2^ total BMD, and Warrington et al.^28^ reported an average 1.049 ± 0.07 g/cm^2^ total BMD among a population of 17 Irish professional flat jockeys. Clearly, many previous studies have ascertained that jockeys have significantly lower BMD that can lead to increased susceptibility to fractures, which could even bring their professional career to an end^2,4,5,26–28,31,42^.

In the present study, the results of correlation analysis between BMD and osteoporosis risk factors showed that only fracture history had a significantly strong correlation. Even though other risk factors did not show significant relationships, future studies that include a larger sample size are needed to corroborate this result.

Horse racing is a high-risk sport in which accidents happen frequently^32–37,48^. All Korean jockeys in this study experienced more than one injury, and two jockeys had more than 10 injuries during an average career span of 11.6 ± 3.8 years. Fractures are the most common type of injury among jockeys. Given the high speed at which horses run in a race and the elevated position of the jockey atop the horse , accidents that occur during a horse race can be very serious and even fatal to jockeys. With the high rate of serious accidents during horse races, low BMD owing to continually poor nutrient consumption can aggravate injuries sustained by jockeys 23. For these reasons, unhealthy dietary habits among jockeys must be rectified and adequate nutritional supplementation, especially of calcium and vitamin D, should be ensured^14,23^.

## CONCLUSION

Korean professional jockeys engage in extreme weight loss practices throughout their careers, and such chronic exposure to undernourished states may result in serious health problems, especially regarding bone health. Given the high rate of accidents in horse racing, the relatively low BMD among jockeys can have very serious consequences. Therefore, efforts should be made to stop and/or regulate repeated extreme weight loss practices among Korean jockeys.
